# Adipokines as biomarkers of postpartum subclinical endometritis in dairy cows

**DOI:** 10.1530/REP-20-0183

**Published:** 2020-06-18

**Authors:** Gonçalo Pereira, Ricardo Bexiga, João Chagas e Silva, Elisabete Silva, Christelle Ramé, Joëlle Dupont, Yongzhi Guo, Patrice Humblot, Luís Lopes-da-Costa

**Affiliations:** 1 CIISA – Centro de Investigação Interdisciplinar em Sanidade Animal, Faculdade de Medicina Veterinária, Universidade de Lisboa, Lisboa, Portugal; 2 INRAE, UMR85 Physiologie de la Reproduction et des Comportements, Nouzilly, France; 3 Division of Reproduction, Department of Clinical Sciences, SLU, Uppsala, Sweden

## Abstract

Adipokines emerged as regulators of metabolism and inflammation in several scenarios. This study evaluated the relationship between adipokines (adiponectin, chemerin and visfatin) and cytological (subclinical) endometritis, by comparing healthy (without), transient (recovered by 45 days postpartum (DPP)) and persistent (until 45 DPP) endometritis cows (*n* = 49). Cows with persistent endometritis had higher adiponectin concentrations in plasma (at 21 DPP, *P* < 0.05 and at 45 DPP, *P* < 0.01) and in uterine fluid (at 45 DPP, *P* < 0.001), and higher chemerin concentrations in plasma (*P* < 0.05) and uterine fluid (*P* < 0.01) at 45 DPP than healthy cows. Cows with persistent endometritis had higher gene transcription in the cellular pellet of uterine fluid and protein expression in the endometrium of these adipokines and their receptors than healthy cows. Adiponectin plasma concentrations allowed to discriminate healthy from persistent endometritis cows, in 87% (21 DPP) and 98% (45 DPP) of cases, and adiponectin and chemerin uterine fluid concentrations at 45 DPP allowed for this discrimination in 100% of cases. Cows with concentrations above the cutoff were a minimum of 3.5 (plasma 21 DPP), 20.4 (plasma 45 DPP), and 33.3 (uterine fluid 45 DPP) times more at risk of evidencing persistent endometritis at 45 DPP than cows with concentrations below the cutoff. Overall, results indicate a relationship between adipokine signalling and the inflammatory status of the postpartum uterus of dairy cows, evidencing that adipokines represent suitable biomarkers of subclinical endometritis, able to predict the risk of persistence of inflammation.

## Introduction

Cow fertility is critical for the sustainable worldwide increasing demand of dairy products and the profitability of the dairy industry (Inchaisri *et al.* 2010, Britt *et al.* 2018). A main factor impairing fertility is the occurrence of postpartum endometritis, which disrupts ovarian and endometrial function leading to a delay in conception and failure in pregnancy establishment (Mateus *et al.* 2002, Sheldon *et al.* 2018). The diagnosis of subclinical endometritis, also termed cytological endometritis, which affects 30–35% of dairy cows between 4 and 9 weeks postpartum (LeBlanc 2008), remains a challenge (Raliou *et al.* 2019). Cytological endometritis is an inflammatory state of the endometrium, detected by histology or cytology, in the absence of purulent vaginal discharge and other clinical signs (Sheldon *et al.* 2006). Due to the invasive nature of the sampling technique, veterinary skills required, time-consuming logistics and cost of the uterine biopsy and swab techniques, the development of reliable, non-invasive biomarkers for the early diagnosis and prognosis of endometritis has been the scope of recent research (Adnane *et al.* 2017, Mayasari *et al.* 2017). The early identification of biomarkers that trigger and/or signal the pathological inflammation of the endometrium would enable to predict uterine health status, administer appropriate prophylactic therapy (Adnane *et al.* 2017) and better manage time of first insemination during the post-partum period.

The adipose tissue serves not only as a depot for lipid storage but also as an endocrine gland that secretes many mediators generally named adipokines (Reverchon *et al.* 2014), including hormone-like mediators as adiponectin, chemerin and visfatin (Kurowska *et al.* 2018). Although adipokines are mainly produced by adipocytes and immune cells found in the stromal vascular fraction of adipose tissue, different cell types outside adipose tissue depots have also been described as primary sources of these mediators (Mancuso 2016, Kurowska *et al.* 2018). Among other functions, adipokines regulate energy metabolism, glucose homeostasis, angiogenesis, reproductive function, immunity and inflammation (Reverchon *et al.* 2014, Mancuso 2016).

Mainly produced by white adipose tissue (Reverchon *et al.* 2014), adiponectin (ADIPOQ) is the most abundant adipokine in human plasma (Barbe *et al.* 2019). Its primary physiological function is to increase insulin sensitivity, but it is also thought that ADIPOQ plays a major role in suppressing systemic and tissue inflammation due to its anti-inflammatory properties (Fang & Judd 2018). Its effects are mainly mediated by two seven-transmembrane domain receptors termed ADIPOR1 and ADIPOR2 (Parker-Duffen *et al.* 2014). These receptors as well as ADIPOQ are expressed in the uterus of humans (Takemura *et al.* 2006), sows (Smolinska *et al.* 2014) and cows (Astessiano *et al.* 2017). In cows, blood ADIPOQ concentrations show a defined pattern, with the nadir around the time of parturition and a progressive increase over the following few weeks of lactation (Barbe *et al.* 2019), and Kasimanickam *et al.* (2013) reported increased ADIPOQ serum concentrations in cows with metritis and clinical endometritis compared to heathy cows.

Chemerin, also known as tazarotene-induced gene 2 protein or retinoic acid receptor responder protein 2 (RARRES2), is a proinflammatory adipokine produced by both adipose tissue and liver (Zabel *et al.* 2014). It is secreted as the inactive precursor prochemerin, which becomes active following cleavage at the C-terminus by extracellular proteases (Mattern *et al.* 2014) and contributes to the regulation of adipogenesis, insulin secretion and the inflammatory process (Kurowska *et al.* 2018). In the latter, RARRES2 works as a chemoattractant for monocytes and dendritic cells (Mancuso 2016), and its blood concentrations correlate with those of TNFα, IL-6 and C-reactive protein (Rourke *et al.* 2013). Chemerin binds to three seven-transmembrane domain receptors – CMKLR1 (Chemokine like receptor 1), CCRL2 (C-C chemokine receptor-like 2) and GPR1 (G Protein-Coupled Receptor 1) (Mariani & Roncucci 2015). The activation of CMKLR1 by RARRES2 induces the migration of macrophages and dendritic cells *in vitro*, which supports the proinflammatory role of the RARRES2/CMKLR1 axis (Yoshimura & Oppenheim 2011). In cows, RARRES2 was referred as a mediator, linking metabolism and ovarian function, and plasma concentrations are positively correlated with body fat mobilization and milk yield (Mellouk *et al.* 2019).

Visfatin, also known as pre-B-cell colony enhancing factor or nicotinamide phosphoribosyl-transferase (NAMPT), is predominantly expressed in visceral adipose tissue but also in muscle, bone marrow, liver, lymphocytes and foetal membranes (Reverchon *et al.* 2014, Dupont *et al.* 2015). Visfatin has an immunomodulatory function and is involved in obesity, inflammation and insulin resistance (Zhang *et al.* 2019). In cows, peripartal serum concentrations of NAMPT were proposed as predictive indicators of retained placenta and other inflammatory diseases (Fadden & Bobe 2016).

Puerperal dairy cows are under metabolic stress, which is related to uterine inflammation (Hammon *et al.* 2006, Guo *et al.* 2019). Owing to the regulation of metabolism and inflammation by adipokines, these molecules may represent promising biomarkers for the early diagnosis and prognosis of postpartum subclinical endometritis. The objective of this study was to evaluate the relationship between adipokines (adiponectin, chemerin, visfatin and their receptors) and postpartum endometritis in dairy cows. The hypothesis behind this study was that adipokine signalling may be active in the cow uterus and that this signalling pattern and adipokine concentrations (blood and uterine) may be related to the inflammatory status of the uterus.

## Materials and methods

### Ethics statement

The experiments were conducted in compliance with the Portuguese legislation for the use of animals for experimental purposes (Decreto-Lei nº129/92 and Portaria nº1005/92, DR nº245, série I-B, 4930-42) and with the European Union legislation (Directive 2010/63/UE). The research protocol was approved by the Institutional Animal Care and Use Committee (Reference CEIE nº37/2019). All animal technical procedures were performed by licenced veterinarians, all of which are accredited as FELASA category C scientists or equivalent.

### Animals, sampling and experimental design

Sample collection was performed in a 400 milking Holstein-Friesian dairy cow herd, with a three daily milking routine and a milk yield average of 11,500 L/cow/305 days of lactation. After calving, cows were monitored daily for signs of puerperal disease. Cows that experienced dystocia, retained foetal membranes, puerperal metritis or mastitis, clinical hypocalcaemia, ketosis and lameness were not included in the study, and enrolled cows did not receive antibiotic or anti-inflammatory therapy throughout the study. Selected cows (*n* = 49) were examined and sampled at 21 (21 ± 0.4) and 45 (44 ± 0.7) days postpartum (DPP). At 21 DPP, cows were blood sampled, the score of the vaginal discharge was assessed, the genital tract was evaluated through transrectal palpation and ultrasonography, and an endometrial swab was taken for cytological examination. At 45 DPP the same procedures were repeated. In addition, as described in details subsequently, a small volume flush and an endometrial biopsy were collected from both uterine horns. Once the voluntary waiting period (50 DPP) was over, cows were submitted to artificial insemination (AI) at the ensuing estrus (detected by visual observation plus pedometer measured walking activity). At the entrance of the milking parlour, an electronic collar identified cows and milk yield and body weight were automatically recorded after each milking. Data including milk yield and body weight (three times daily), estrus and insemination dates, and pregnancy status (at 35–42 and 90 days post-AI) were retrieved from the herd management software, until completion of lactation (305 DPP).

The experimental design considered the comparison between three groups, with cows being retrospectively allocated to: (1) control healthy cows, without cytological endometritis at 21 and 45 DPP (group HH; *n* = 19); (2) cows with cytological endometritis at 21 DPP that had recovered by 45 DPP (group EH; *n* = 19); and (3) cows with cytological endometritis at 21 DPP, which persisted at 45 DPP (group EE; *n* = 11). A further refinement of group HH considered two subsets of cows, including cows pregnant at first AI (subset HHP; *n* = 6) and cows pregnant at subsequent AIs (*n* = 13). The subset HHP was considered for ‘extreme cases’ comparison (healthy pregnant vs endometritis) in endometrial immunolocalization and transcription analysis of the cellular pellet of uterine flushing.

### Blood sampling

Blood samples were aseptically collected by venipuncture of the coccygeal vein into 10 mL dry tubes and 10 mL tubes containing K3 EDTA (13060, Vacutest KIMA, Arzegrande, Italy). Tubes were immediately centrifuged (2000 ***g*** for 15 min), plasma and serum were aliquoted into 1.5 mL Eppendorfs and stored at −20°C until subsequent analysis for adipokines, non-esterified fatty acids (NEFA) and progesterone concentrations.

### Genital tract evaluation

The genital tract was palpated per-rectum to assess the size of the cervix and uterus and the symmetry and tonus of uterine horns. An ensuing ultrasound examination (ExaGo, Echo Control Medical, France) evaluated the uterine content (intrauterine fluid volume and echogenicity) and identified and measured the ovarian structures. The vaginal discharge score was graded in a 0–3 scale according to Williams *et al.* (2005), following collection with a Metricheck device (EndoControl Sampler, Minitube, La Selva del Camp, Spain).

### Endometrial cytology

Endometrial swabs were performed using an adapted cytobrush technique. A cervical brush (Bastos Viegas SA, Penafiel, Portugal) was aseptically adapted to the inner stylet of and retracted into an AI gun (IMV technologies, L’Aigle, France) covered with an AI sheath (IMV technologies), and the swab obtained as described by Pascottini *et al.* (2016). The brush was then gently rolled along the length of two glass microscope slides, which were subsequently labelled and air-dried. Slides were stained with a modified Wright-Giemsa® stain (Diff-Quick, MAIM SL, Barcelona, Spain) and the percentage of polymorphonuclear neutrophils (PMN) was assessed from 400 cells (200 in each slide). At 21 DPP a ≥18% PMN cut-off was chosen (Kasimanickam *et al.* 2004), and cows with a PMN percentage below this cut-off were considered without cytological endometritis. At 45 DPP, a ≥5% PMN cut-off was chosen (Gilbert *et al.* 2005) and, similarly, cows with a PMN percentage below this cut-off were considered without cytological endometritis.

### Uterine flushing

Both uterine horns were flushed independently with sterile PBS (Millipore Corp), using a sterile silicone-coated latex Foley catheter (Rusch Gold, Rusch, Perak, Malaysia) aseptically mounted in a metal stylet and covered with a sanitary sheath (IMV technologies). At the cervical external opening, the sanitary sheath was retracted, the catheter guided to one of the uterine horns past the external bifurcation level, and the cuff inflated. Fifty milliliters of PBS were infused into each uterine horn, which was gently massaged per-rectum, before allowing collection of the fluid into a 50 mL centrifuge tube. Samples were transported to the laboratory at 4°C, filtered with a mesh to remove mucus and debris, centrifuged (5000 ***g*** for 15 min), and the supernatant and cellular pellet independently stored at −80°C until assayed, respectively, for adipokines’ concentrations and transcription of adipokine signalling genes.

### Endometrial biopsy

Endometrial biopsies were independently collected from both uterine horns by using Kervokian–Younge endometrial biopsy instrument (Alcyon, Paris, France), according to procedures described by Chapwanya *et al.* (2009, 2010). Briefly, a low epidural anaesthesia was performed with 200 mg of Procaine hydrochloride + 0.18 mg of Epinephrine tartrate (Pronestesic, Fatro, Bologna, Italy) injected in the epidural cavity at the first coccygeal space. After hygiene of the vulva, the sterile biopsy instrument, guarded in a protective sheath, was introduced into the vagina and advanced to the external cervical orifice, where the protective sheath was ruptured. The biopsy instrument was then guided into the first third of the uterine horn and an endometrial sample of about 1.5 cm^2^ was recovered. The endometrial sample was cut into three equal size portions, one of which was immediately fixed in 4% paraformaldehyde (PFA) and paraffin embedded within 24–48 h for immunohistochemistry analysis. The remaining portions were processed for other analysis.

### Progesterone assay

Serum progesterone was assayed by a chemiluminescent immunoassay in an IMMULITE 1000 analyser (Siemens Healthcare Diagnostics), using a commercial kit (IMMULITE 1000 Progesterone Kit, Siemens Healthcare Diagnostics). The analytical sensitivity of the assay was 0.2 ng/mL and the inter-assay coefficient of variation was <10%. Cows were classified as in luteal phase (at 21 and 45 DPP) when progesterone concentration was >0.5 ng/mL and an ovarian luteal structure (corpus luteum) was detected during the ultrasound examination.

### Non-esterified fatty acids assay

Serum NEFA concentrations were determined by a colorimetric method (kit no. FA 115, Randox, Crumlin, UK) using a Randox RX Daytona equipment (Randox). Each sample was analysed in duplicate and the quality control of the assay was performed with the Randox Acusera Assayed Chemistry Premium Plus Control (Assayed Chemistry Premium Plus Level 2 and 3, Randox). The analytical sensitivity of the assay was 0.072 mmol/L and the inter-assay coefficient of variation was <5%.

### Adipokine assays

Plasma and uterine fluid total ADIPOQ, RARRES2 and NAMPT concentrations were measured by using ELISA kits (E11A0125 and E11C0104 from BlueGene, Shanghai, China and EK-003-80 from Phoenix France, SAS, Strasbourg, France, respectively), as previously described (Fadden & Bobe 2016, Mellouk *et al.* 2017, 2019). The intra-assay coefficients of variation for total ADIPOQ, RARRES2 and NAMPT were 6%, 4.5% and 6.5%, respectively. The inter-assay coefficients of variation for total ADIPOQ, RARRES2 and NAMPT were 6.2%, 6.5% and 6%, respectively. Adipokine uterine fluid measurements (as well as quantitative real time RT-PCR of the uterine fluid cell pellet; see subsequently) were performed in samples recovered from the uterine horn contralateral to the corpus luteum or to a randomly selected uterine horn if no corpus luteum was present.

### Immunohistochemistry

Endometrial samples from three cows of each group (HHP, EH and EE) were used for protein immunolocalisation. Paraffin embedded endometrial samples were serially sectioned at a thickness of 4 μm, deparaffinised and hydrated. Quenching of endogenous peroxidase activity, antigen retrieval (EnVision™ FLEX Target Retrieval Solution, DM829; Dako, Glostrup, Denmark) and nonspecific background elimination were performed according to Maillard *et al.* (2010). Twin sections were incubated overnight at 4°C with control rabbit or mouse IgG and the different primary antibodies (Supplementary Table 1, see section on supplementary materials given at the end of this article) diluted in PBS with 5% lamb serum. Sections were then washed four times in PBS for 10 min and incubated for 30 min at room temperature with a ready-to-use labelled polymer-HRP (EnVision™ FLEX/HRP detection reagent, SM802; Dako). After washing twice in PBS for 10 min, immunoreactivity was revealed by incubation with DAB chromagen/DAB buffer at room temperature, according to manufacturer instructions (Envision™ FLEX, K8000; Dako). Finally, the slides were counterstained with Meyers haematoxylin (Merck), then dehydrated and mounted with Entellan® (Merck). Staining intensity and cell type identification were independently assessed by two observers, at 100× and 1000× magnification. Intensity was classified as – (absent); +/− (weak); + (moderate); ++ (strong). From each cow, staining was assessed from a minimum of three optical fields from twin slides for each antibody.

### Quantitative real time RT-PCR analysis of *ADIPOQ*, *ADIPOR1*, *ADIPOR2*, *RARRES2*, *CMKLR1* and GPR1 in the cellular pellet of the uterine flushing

Total RNA of the cellular pellet of the uterine flushing performed at 45 DPP was extracted using the RNeasy Midi kit (Qiagen®), according to manufacturer instructions. Concentration and purity of RNA were determined with a NanoDrop Spectrophotometer (Peqlab Biotechnologie GmbH, Erlangen, Germany), RNA integrity evaluated on 1.25% agarose-formaldehyde gels, and total RNA quality assessed with an Agilent Bioanalyzer 2100, using a RNA 6000 pico kit (Agilent Technologies). The 260/280 ratio (1.7–1.9) and RIN value (7.2–8.5) evidenced a suitable RNA quality for analysis (Supplementary Table 2). The cDNA was generated by RT of total RNA (1 μg) in a mixture comprising 0.5 mM of each deoxyribonucleotide triphosphate (dATP, dGTP, dCTP, DTTP), 2 M of RT buffer, 15 μg/μL of oligodT, 0.125 U of ribonuclease inhibitor, and 0.05 UMMLV (Moloney murine leukemia virus reverse transcriptase) for 1 hour at 37°C. Real-time PCR was performed using the MyiQ Cycle device (Bio-Rad), in a mixture of SYBR Green Supermix 1X reagent (Bio-Rad), 250 nM specific primers (Invitrogen™ by Life Technologies™) (Supplementary Table 3) and 5μL of cDNA diluted 1:5, in a total volume of 20 μL. The amplification protocol started with an incubation for 2 min at 50°C and a denaturation step of 10 min at 95°C, followed by 40 cycles (30 s at 95°C, 30 s at 60°C, 30 s at 72°C), and acquisition of the melting curve. Each sample was analysed in duplicate in the same plate, and amplification with water, instead of cDNA, was performed systematically as a negative control. Gene transcription was calculated according to primer efficiency (E) and quantification cycle (Cq), where transcription *n* = E − Cq. Then, relative expression of the target gene was analyzed following standardization of the level of mRNA expression for the geometric mean of three reference genes (*GAPDH*, *ACTB* and *PPIA*) which were reported as accurate normalisation factors (Vandesompele *et al.* 2002).

### Statistical analysis

Data were managed in Microsoft Excel and statistical analyses were conducted using SPSS for Windows 26.0 (IBM Corp.). The normality of the distribution of the data was tested with the Shapiro–Wilk-Test. Values are reported as median and interquartile range for non-normally distributed variables and mean ± s.e.m. for those normally distributed (Supplementary Table 4). Significant differences between groups were determined using a one-way ANOVA or the non-parametric Kruskal–Wallis-Test with Dunns post-test and Bonferroni correction for multiple tests, respectively, for normally and non-normally distributed variables. The level of significance was set at *P* < 0.05. Correlation analysis used the Spearman’s rank test. The predictive accuracy of the different adipokines to discriminate cows with cytological endometritis was evaluated by Receiver Operating Characteristic (ROC) curve analysis, testing the probability (P) that the area under curve (AUC) differed significantly from random (AUC = 0.5). ROC curve coordinates allowed Youden Index calculation, best cut-off and odds ratio (OR) determination for ADIPOQ and RARRES2 concentrations. Cohen’s kappa statistic was used to assess the agreement between the vaginal discharge Metricheck score and the endometrial cytology PMN percentage.

## Results

### Endometrial cytology PMN percentage and vaginal discharge Metricheck score

Endometrial cytology PMN percentage reflects the retrospective allocation of cows to groups, based on endometrial cytology cut-off (Table 1). At 21 DPP, the endometrial cytology PMN percentage was lower in group HH than in groups EH and EE (*P* < 0.001). However, at 45 DPP, the endometrial cytology PMN percentage was similar in groups HH and EH and lower than in group EE (*P* < 0.001). At 21 DPP, the vaginal discharge Metricheck score was lower (*P* = 0.001) in group HH than in group EE, but at 45 DPP, the vaginal discharge Metricheck score was similar in all groups. At 21 DPP, there was a moderate level of agreement (Cohen’s Kappa = 0.47; *P* < 0.01) between the vaginal discharge Metricheck score and the endometrial cytology PMN percentage (Supplementary Table 5A). However, at 45 DPP, there was no agreement between the mentioned parameters (Cohen’s Kappa = −0.10; *P* = 0.46) (Supplementary Table 5B).

**Table 1:** Metabolic and fertility parameters in healthy cows (group HH), cows with cytological endometritis at 21 DPP but that recovered by 45 DPP (group EH), and cows with persistent cytological endometritis until 45 DPP (group EE).

Parameters	HH (*n* = 19)	EH (*n* = 19)	EE (*n* = 11)
Lactation number*	1 (1–2)	2 (1–2)	2 (1–3)
Body Weight Loss Calving – 21 DPP (%)*	2.2 (0–5.4)	1.9 (−1.2–5.8)	7.5 (−0.2–9.0)
Body Weight Loss Calving – 45 DPP (%)*	4.1 ± 1.8	2.5 ± 1.4	3.9 ± 1.8
Body Weight Loss Calving – 60 DPP (%)*	3.6 (1.6–7.8)	1.3 (−1.9–5.0)	4.9 (−1.1–10.2)
Milk yield by 21 DPP (L)**	863.5 ± 38.7	839.8 ± 33.0	874.0 ± 48.6
Milk yield by 45 DPP (L)**	1716.8 ± 76.5	1710.0 ± 64.7	1806.7 ± 95.0
Milk yield by 60 DPP (L)**	2375.2 ± 102.1	2392.6 ± 88.0	2512.4 ± 133.7
NEFA at 21 DPP (mmol/L)*	0.28 (0.17–0.50)	0.25 (0.17–0.60)	0.26 (0.20–0.43)
NEFA at 45 DPP (mmol/L)*	0.27 (0.17–0.50)	0.28 (0.14–0.43)	0.17 (0.13–0.34)
Cows in luteal phase at 21 DPP (*n* (%))	14 (74)^a^	5 (26)^b^	6 (55)^a,b^
Cows in luteal phase at 45 DPP (*n* (%))	18 (89)^a^	10 (53)^b^	7 (64)^b^
Metricheck Score at 21 DPP*	0 (0–1)^a^	1 (0–2)^a,b^	2 (2–2)^b^
Metricheck Score at 45 DPP*	0 (0–0)	0 (0–0)	0 (0–0)
PMN % in Cytobrush at 21 DPP*	4.0 (1.0–8.0)^a^	25.5 (20–41.5)^b^	61 (35–86)^b^
PMN % in Cytobrush at 45 DPP*	1 (0.0–1.0)^a^	1 (0–1)^a^	11 (7–17)^b^
Calving – first AI interval (days)*	61 (56–67)	61 (58–80)	64 (50–94)
Cows pregnant at first AI (*n* (%))	6 (32)	0	0
Cows conceiving within 305 DPP (*n* (%))	19 (100)	14 (74)	7 (64)
Calving – conception interval (days)*	136 (64–232)	110 (98–157)	94 (85–236)
*n*	19	14	7
AI number/conception* *n*	4 (1–6) 19	3 (2–4) 14	2 (2–7) 7

Different letters indicate significant differences between groups (level of significance *P* < 0.05) determined with ANOVA and the non-parametric Kruskal–Wallis test with Dunns post-test for normally and non-normally distributed variables, respectively.

*Values reported as median and (interquartile range) for non-normally distributed data; **Values reported as mean ± s.e.m. for normally distributed data.

DPP, days postpartum.

### Metabolic and fertility parameters

As shown in Table 1, at 21 DPP and 45 DPP, there were no significant differences between groups in mean milk yield, body weight loss and serum NEFA concentrations. There were no significant differences for the calving to first AI interval, calving to conception interval and AI number/conception between the three groups. However, the latter two parameters of groups EH and EE were favoured by only including pregnant cows within 305 DPP (as five and four cows were culled for non-conception at 305 DPP, respectively). Six of 19 (32%) HH cows conceived at first AI and the remaining were pregnant within 305 DPP. Also, at 45 DPP there were more cows in luteal phase in group HH than in groups EH and EE (*P* = 0.01), evidencing an earlier resumption of ovarian activity of HH cows, compared to EH and EE cows.

### Adipokine concentrations in plasma and uterine fluid

**Figure 1:**
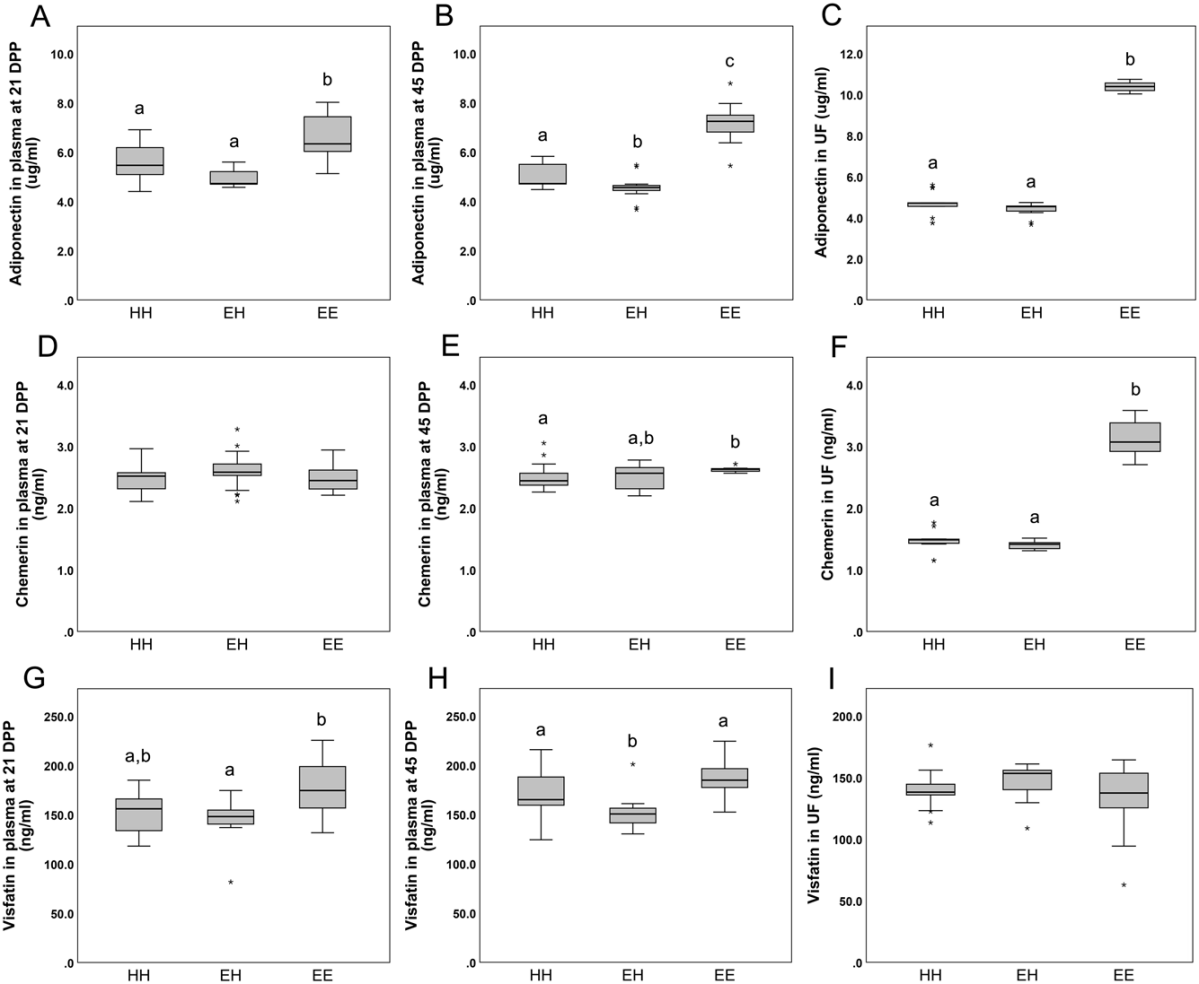
Plasma and uterine fluid concentrations of adiponectin (A, B and C), chemerin (D, E and F) and visfatin (G, H and I) in healthy cows (group HH), cows with cytological endometritis at 21 DPP but recovered by 45 DPP (group EH), and cows with persistent cytological endometritis until 45 DPP (group EE). Different letters indicate a significant difference at *P* < 0.05. Horizontal black lines indicate median, boxes extend from the 25th to the 75th percentile and vertical lines indicate values within 1.5 interquartile range of the 25th and 75th percentile. Asterisks indicate outliers.

Plasma and uterine fluid concentrations of ADIPOQ, RARRES2 and NAMPT are presented in Fig. 1 and Supplementary Table 6. ADIPOQ concentrations in plasma at 21 (*P* < 0.05) and 45 DPP (*P* < 0.01) and uterine fluid (*P* < 0.001) were higher in EE cows than in HH and EH cows. At 45 DPP, plasma concentrations of RARRES2 were higher (*P* < 0.05) in EE than in HH cows and uterine fluid concentrations were higher (*P* < 0.01) in group EE than in groups HH and EH. In contrast, uterine fluid concentrations of NAMPT were similar in the three groups. Nevertheless, plasma NAMPT concentrations of EH cows were lower (*P* < 0.01) than of EE cows at 21 DPP and of both HH and EE cows at 45 DPP (*P* < 0.01). Plasma concentrations of ADIPOQ, RARRES2 and NAMPT at 21 DPP and 45 DPP were similar in luteal phase and follicular/anoestrous cows of groups HH, EH and EE. This was also observed in uterine concentrations at 45 DPP, except in HH group, where only ADIPOQ concentrations were higher (*P* < 0.01) in luteal than in follicular/anoestrous cows (data not shown).

### Relationship between adipokines’ concentrations and PMN percentage from endometrial cytology

Table 2 displays a summary of Spearman correlations between adipokines’ concentrations and PMN percentage from endometrial cytology. Uterine fluid concentrations of ADIPOQ and RARRES2 showed a significant positive correlation with the endometrial cytology PMN percentage at 45 DPP. In addition, concentrations of ADIPOQ in plasma significantly correlated with those in uterine fluid and at 45 DPP with the endometrial cytology PMN percentage. RARRES2 uterine fluid levels did not correlate with its plasma levels at 45 DPP.

**Table 2:** Spearman’s correlations between the plasma (21 and 45 DPP) and uterine fluid (UF; 45 DPP) adipokines’ concentrations and the PMN percentage from endometrial cytology (*n* = 49).

	ADIPOQ 21 DPP	ADIPOQ 45 DPP	ADIPOQ UF	RARRES2 45 DPP	RARRES2 UF	PMN % 21 DPP	PMN % 45 DPP
ADIPOQ 21 DPP		0.599**	0.631**	0.349*	0.457**	0.087^ns^	0.281^ns^
ADIPOQ 45 DPP			0.704**	0.216^ns^	0.720**	0.087^ns^	0.436**
ADIPOQ UF				0.093^ns^	0.782**	0.244^ns^	0.537**
RARRES2 45 DPP					−0.019^ns^	0.231^ns^	0.153^ns^
RARRES2 UF						0.218^ns^	0.640**
PMN % 21 DPP							0.507**

*P < 0.05 and **P < 0.01.

DPP, days postpartum; ^ns^, not significant.

**Figure 2:**
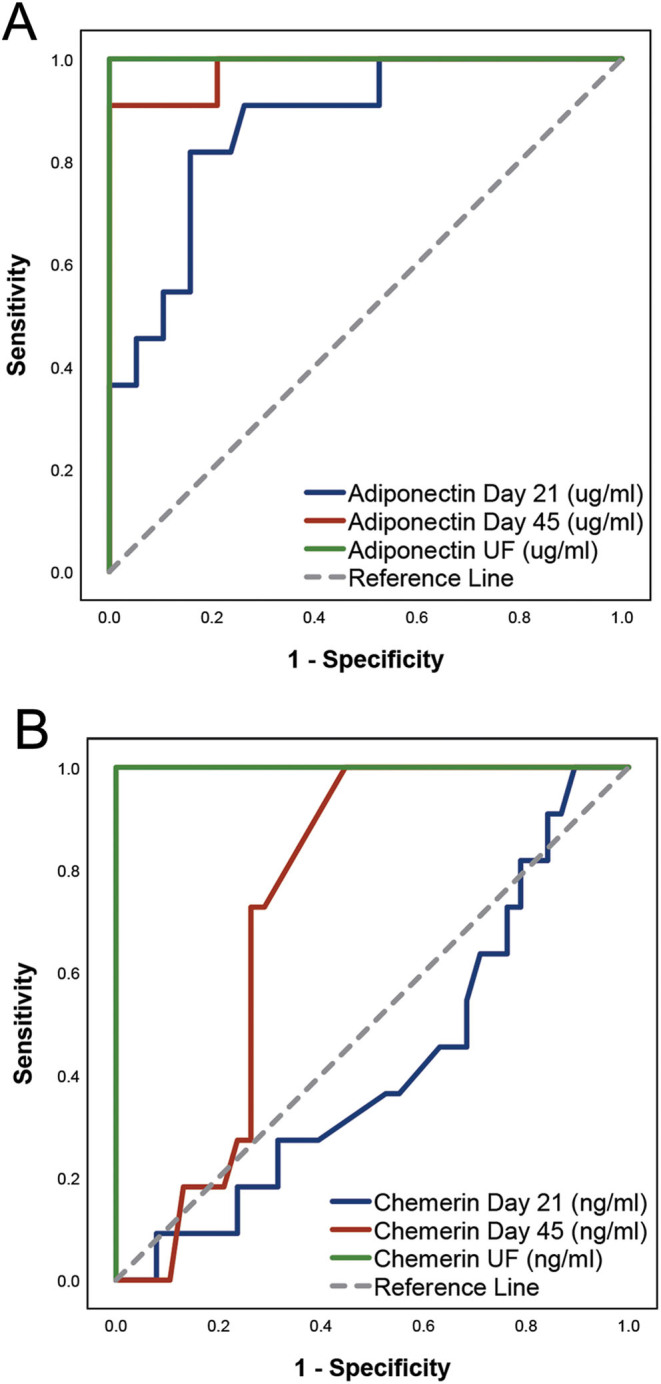
Receiver operating characteristic curve analysis of adiponectin (A) and chemerin (B) to discriminate cows with cytological endometritis at 45 days postpartum. Concentrations were measured in plasma at 21 and 45 days postpartum and in uterine fluid (UF) at 45 days postpartum.

Figure 2 represents the ROC analysis of ADIPOQ and RARRES2 concentrations, to discriminate cows with cytological endometritis at 45 DPP. Plasma ADIPOQ at 21 DPP (AUC = 0.872; *P* < 0.001), plasma ADIPOQ at 45 DPP (AUC = 0.981; *P* < 0.001), and uterine fluid ADIPOQ and RARRES2 at 45 DPP (AUC = 1.000; *P* < 0.001) showed high AUC values. In contrast, plasma RARRES2 at 21 DPP (AUC = 0.423; *P* = 0.443) and at 45 DPP (AUC = 0.738; *P* = 0.017) showed either no ability or a low ability to discriminate cows with cytological endometritis. The risk of endometritis persistence at 45 DPP (Supplementary Table 7) was higher for cows with plasma ADIPOQ concentrations at 21 DPP above 5.9 µg/mL (OR = 19.9; 95% CI: 3.5–113.3; *P* < 0.001) than for cows with concentrations below the cutoff. At 45 DPP, plasma and uterine fluid ADIPOQ concentrations showed a high discriminatory power for diagnosis of endometritis. Cows with plasma and uterine fluid ADIPOQ concentrations above 6.1 and 7.8 µg/mL, respectively, had increased risk of being diagnosed with endometritis (OR = 539; 95% CI: 20.4–14,218.7; *P* < 0.001 and OR = 1771; 95% CI: 33.3–94,301.3; *P* < 0.001, respectively) than cows with concentrations below the cutoff. For plasma concentrations, only 1 of 39 cows with concentrations below the cutoff showed endometritis and all 10 cows with concentrations above the cutoff presented endometritis. For uterine fluid concentrations, all cows with concentrations below the cutoff were healthy, whereas all cows with concentrations above the cutoff presented endometritis. This was also observed in the case of uterine fluid RARRES2 concentrations (cutoff = 2.2 ng/mL).

As concentrations in plasma and uterine fluid showed significant differences between healthy (HH) and endometritis cows (EE) only for ADIPOQ and RARRES2, the analysis of the endometrial immunolocalisation and of the gene expression in cellular pellets from uterine flushings was performed only for these two adipokines and their receptors. Also, to further refine the evaluation of differences between healthy and endometritis cows (’extreme cases’), the analysis considered a subset of the HH group, consisting of healthy cows that became pregnant at first AI (group HHP; *n* = 6).

### Immunolocalisation of ADIPOQ, ADIPOR1, ADIPOR2, RARRES2 and CMKLR1 in the bovine endometrium

**Figure 3:**
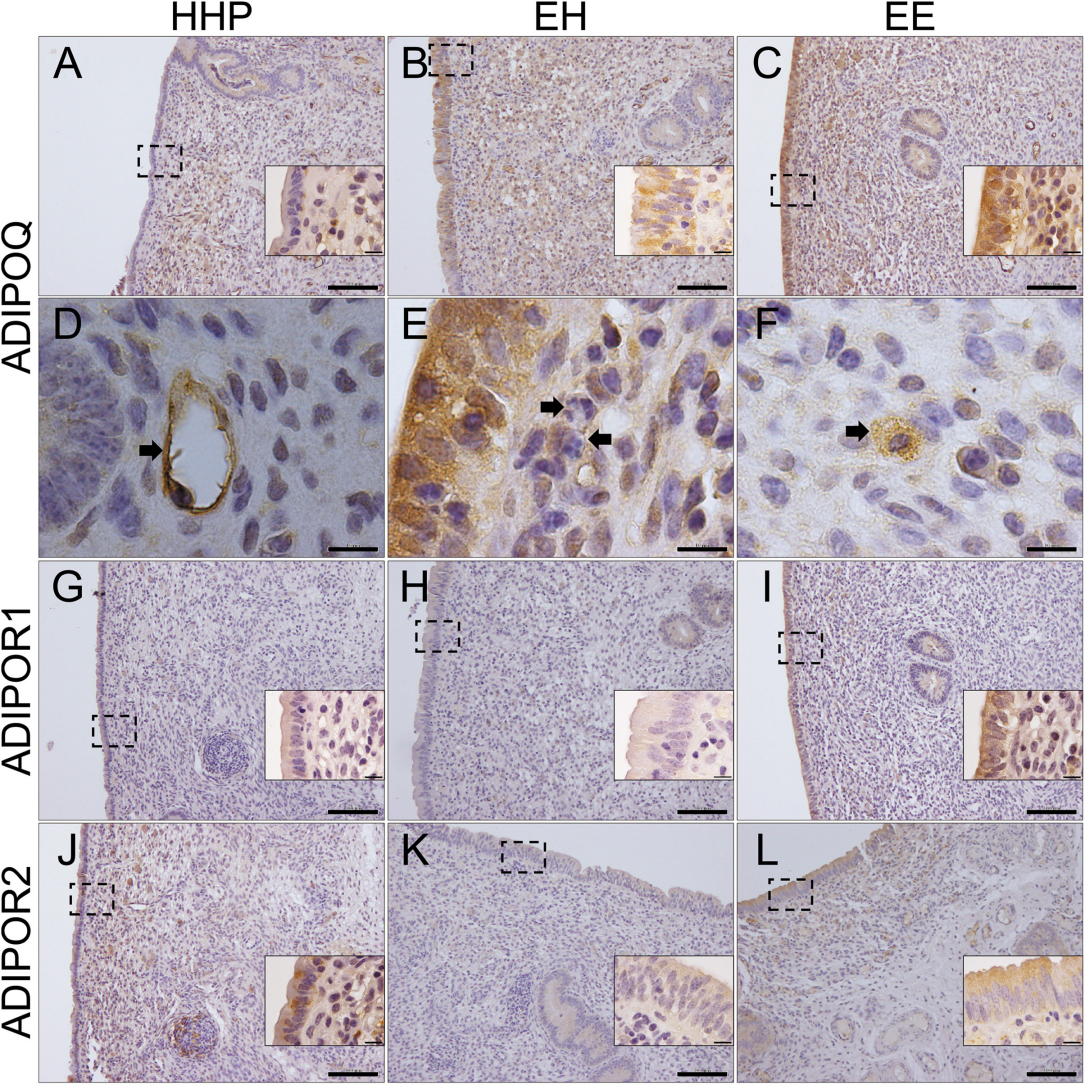
Representative photomicrographs of endometrial sections from biopsies recovered at 45 days postpartum from a subset of healthy cows pregnant at first AI (group HHP), cows with cytological endometritis at day 21 postpartum but that recovered by day 45 postpartum (group EH), and cows with persistent cytological endometritis until day 45 postpartum (group EE). Immunostaining for ADIPOQ (A, B, C, D, E and F), for ADIPOR1 (G, H and I) and for ADIPOR2 (J, K and L). In D, arrow pointing to stained endothelial cell; in E, arrows pointing to non-stained PMN; in F, arrow pointing to stained macrophage. Scale bar 10 µm (D, E, F and all the insets) and 100 µm (A, B, C, G, H, I, J, K and L).

**Figure 4:**
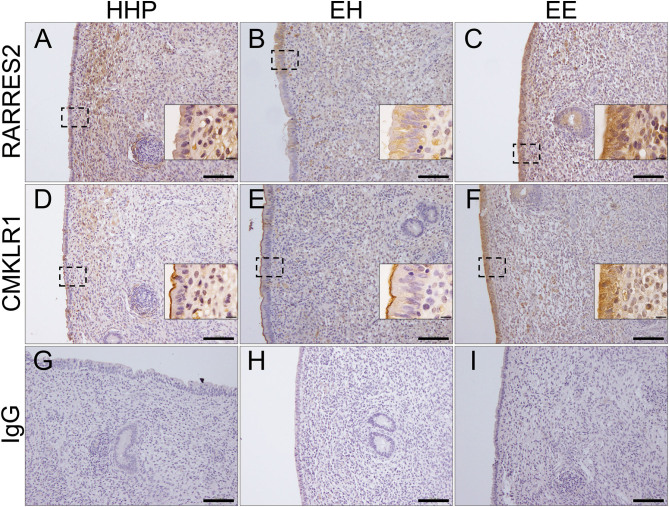
Representative photomicrographs of endometrial sections from biopsies recovered at 45 days postpartum from a subset of healthy cows pregnant at first AI (group HHP), cows with cytological endometritis at day 21 postpartum but that recovered by day 45 postpartum (group EH), and cows with persistent cytological endometritis until day 45 postpartum (group EE). Immunostaining for RARRES2 (A, B, C), CMKLR1 (D, E, F) and negative control (G, H, I). Scale bar 10 µm (insets) and 100 µm (A, B, C, D, E, F, G, H and I).

Positive immunostaining for ADIPOQ was observed in the luminal and glandular epithelial cells and in endothelial, stromal and inflammatory cells of all cows. However, cows of group EE exhibited a stronger staining in the luminal epithelium than cows of HHP and EH groups (Fig. 3A, B, C and Table 3). Positive staining for ADIPOQ was observed in endothelial cells (Fig. 3D) and macrophages (Fig. 3F) within the stroma, whereas the infiltrated PMN were negative (Fig. 3E). Positive immunostaining for ADIPOR1 and ADIPOR2 was observed in luminal and glandular epithelial cells. Additionally, ADIPOR2 staining was observed in stromal and inflammatory cells (Fig. 3G, H, I, J, K and L). Both these receptors displayed a stronger staining in EE cows than in HHP and EH cows (Table 3). Positive immunostaining for RARRES2 was detected in the luminal and glandular epithelia and in stroma and inflammatory cells of all cows (Fig. 4A, B and C), and the level of staining was stronger in EE group than in HHP and EH groups (Table 3). Positive immunostaining for CMKLR1 was observed in the luminal epithelium and in stroma and inflammatory cells (Fig. 4D, E and F). However, in the luminal epithelium of HHP and EH cows, the staining was mostly found in the apical membrane of the cells (Fig. 4D and E), whereas in EE cows a strong staining is present throughout the cytoplasm (Fig. 4F).

**Table 3:** Level of immunostaining of ADIPOQ, ADIPOR1, ADIPOR2, RARRES2 and CMKLR1 in different cell populations of endometrial biopsies taken at 45 DPP in healthy cows (group HHP, *n* = 3), cows with cytological endometritis at 21 DPP but that recovered by 45 DPP (group EH, *n* = 3), and cows with persistent cytological endometritis until 45 DPP (group EE, *n* = 3).

	HHP	EH	EE
ADIPOQ			
LE	+/−	+	++
GE	+	+	+
ST	+	+	+
ST-IC	+/−	+	+
ST-LF	-	-	+/−
ADIPOR1			
LE	+/−	+	++
GE	+	+	+
ST	-	-	-
ST-IC	-	-	-
ST-LF	-	-	-
ADIPOR2			
LE	+/−	+	++
GE	+	+	+
ST	+	+	+
ST-IC	+	+	+
ST-LF	+	+/−	+
RARRES2			
LE	+	+	++
GE	+	+/−	+
ST	+	+	+
ST-IC	+	+	+
ST-LF	+	+/−	+/−
CMKLR1			
LE	+	+	++
GE	-	-	-
ST	+/−	+/−	+
ST-IC	+/−	+/−	++
ST-LF	+/−	-	+/−

DPP, days postpartum; GE, glandular epithelium; LE, luminal epithelium; level of staining: - (absent), +/− (weak), + (moderate), ++ (strong); ST, stroma cells; ST-IC, inflammatory cells in stroma; ST-LF, lymphoid follicles in stroma.

### Transcription levels of *ADIPOQ*, *ADIPOR1*, *ADIPOR2*, *RARRES2*, *CMKLR1*, *GPR1* and* CCRL2* in the cellular pellet of the uterine flushing

**Figure 5:**
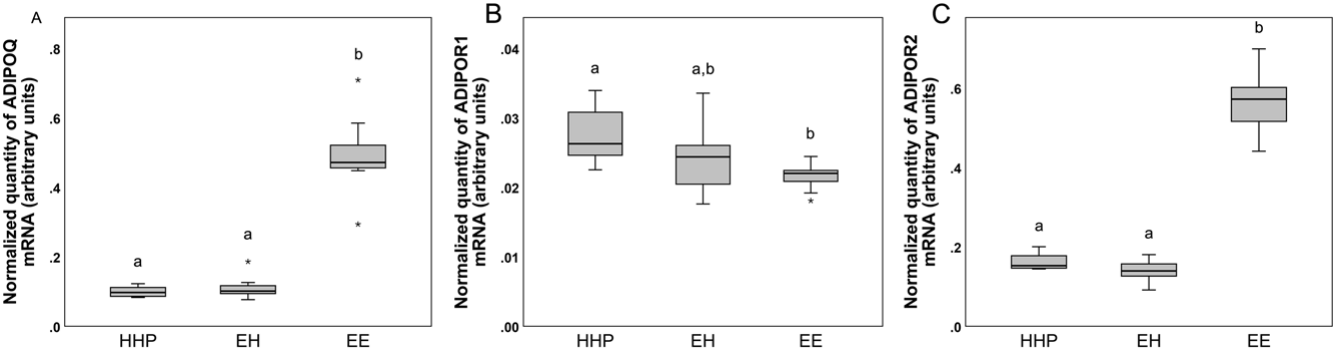
Transcript abundance of *ADIPOQ* (A), *ADIPOR1* (B) and *ADIPOR2* (C) standardized to the geometric mean of *GAPDH*, *ACTB* and *PPIA* in uterine cell pellets collected at 45 days postpartum. Data analysed using Kruskal–Wallis test with Dunns post-test. Different letters indicate a significant difference at *P* < 0.05. Groups HHP, healthy cows pregnant at first AI; EH, cows with cytological endometritis at 21 days postpartum but that recovered by 45 days postpartum; EE, cows with persistent cytological endometritis until 45 days postpartum. Horizontal black lines indicate median, boxes extend from the 25th to the 75th percentile and vertical lines indicate values within 1.5 interquartile range of the 25th and 75th percentile. Asterisks indicate outliers.

**Figure 6:**
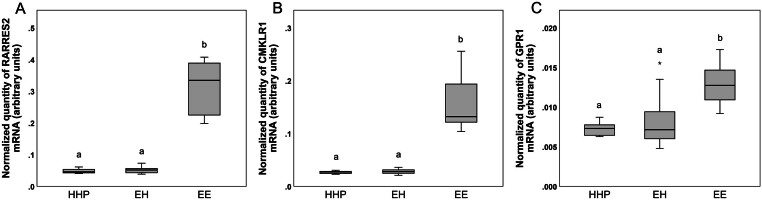
Transcript abundance of RARRES2 (A), CMKLR1 (B) and GPR1 (C) standardized to the geometric mean of GAPDH, ACTB and PPIA in uterine cell pellets collected at 45 days postpartum. Data analysed using Kruskal–Wallis test with Dunns post-test. Different letters indicate a significant difference at *P* < 0.05. Groups HHP, healthy cows pregnant at first AI; EH, cows with cytological endometritis at 21 days postpartum but that recovered by 45 days postpartum; EE, cows with persistent cytological endometritis until 45 days postpartum. Horizontal black lines indicate median, boxes extend from the 25th to the 75th percentile and vertical lines indicate values within 1.5 interquartile range of the 25th and 75th percentile. Asterisks indicate outliers.

Transcription levels of *ADIPOQ*, *ADIPOR1*, *ADIPOR2*, *RARRES2*, *CMKLR1* and *GPR1* are presented in Figs 5 and 6. Transcription levels of *ADIPOQ* and *ADIPOR2* were higher (*P* < 0.001) in group EE than in groups HHP and EH. In contrast, mRNA levels of* ADIPOR1* were lower (*P* < 0.05) in EE cows than in HHP cows. Transcription levels of *RARRES2*, *CMKLR1* and *GPR1* were also higher (*P* < 0.001, *P* < 0.001 and *P* = 0.001, respectively) in group EE than in groups HHP and EH, whereas transcription levels of *CCRL2* were similar in the three groups (data not shown).

## Discussion

There is a wide range in the reported prevalence of cytological endometritis in dairy cows, but it is frequently pointed around 30–35% between 4 to 9 weeks postpartum (LeBlanc 2008). Here, in cows without puerperal disease, the prevalence of cytological endometritis was 61% (30 out of 49) at 21 DPP and 22% (11 out of 49) at 45 DPP. This shows that over 60% of early affected cows spontaneously recovered by the end of the voluntary waiting period exhibiting transient endometritis. This also shows that, from the total number of cows initially recruited at 21 DPP, 22% still suffer from persistent endometrial inflammation at 45DPP.

At 21 DPP, 83% of cows with cytological endometritis showed a positive vaginal discharge Metricheck score, indicating the subclinical nature of endometrial inflammation in about 20% of cows. In contrast, at 45 DPP, only 9% of cows with cytological endometritis showed a positive vaginal discharge Metricheck score, indicating the subclinical nature of endometritis in about 90% of cows. These figures, as reported by McDougall *et al.* (2011), demonstrate a moderate level of agreement between the vaginal discharge Metricheck score and the endometrial cytology PMN percentage at 21 DPP and no agreement at 45 DPP. This may be partially explained by or may reflect the presence of cervicitis or vaginitis instead of endometritis (Westermann *et al.* 2010). By contrast, the onset of postpartum ovarian activity promotes cervix closure under the influence of progesterone, which may block the flux of uterine contents into the vagina.

Therefore, for descriptive purposes, herein cows with cytological endometritis until 45 DPP are also referred as persistent (cytological) endometritis cows, whereas cows that recovered of cytological endometritis by 45 DPP are also referred as transient (cytological) endometritis cows.

Milk yield, body weight loss and blood NEFA concentrations were not associated to persistent or transient cytological endometritis. These findings are in agreement with Dubuc *et al.* (2011) and reinforce the subclinical nature of this condition. In dairy cows, plasma concentrations of adipokines are affected by negative energy balance status (Mellouk *et al.* 2017), variations in white adipose tissue quantities (Reverchon *et al.* 2014) and milk yield (Kafi *et al.* 2015). However, no differences were observed here between uterine health groups regarding these parameters. Therefore, it is unlikely that they have contributed to the observed between-group differences in adipokines’ gene transcription, protein expression and production described subsequently.

This study evidenced that cows affected by persistent endometritis exhibited increased concentrations of ADIPOQ at 21 and 45 DPP in plasma and at 45 DPP in uterine fluid, compared to healthy and transient endometritis cows. The ROC curve analysis showed that the chance of discriminating a healthy from a persistent endometritis cow, at 45 DPP, was 87% and 98% based on plasma concentrations at 21 and 45 DPP, respectively, and 100% based on uterine fluid concentrations at 45 DPP. In addition, the OR analysis showed that cows with concentrations above the cutoff were a minimum of 3.5 (plasma 21 DPP), 20.4 (plasma 45 DPP) and 33.3 (uterine fluid 45 DPP) times more at risk of evidencing persistent endometritis at 45 DPP than healthy cows. These observations evidence that ADIPOQ is a promising biomarker, with putative high discriminatory power for the identification of persistent endometritis cows at 45 DPP. In contrast with these results, Kasimanickam *et al.* (2013), although reporting increased concentrations of serum ADIPOQ in cows affected by metritis and clinical endometritis, found no differences between cows affected by subclinical endometritis and healthy cows. However, the study (Kasimanickam *et al.* 2013) only included six subclinical endometritis cows, and four cows of the healthy cow group (*n* = 13) were subsequently found to develop clinical endometritis 2 weeks later. Therefore, the inconsistency between the two studies may be explained by differences in population size and categorization of the healthy cow group.

The increased plasma and uterine fluid ADIPOQ concentrations observed in endometritis cows can result from white adipose tissue production or it can originate from local expression in the reproductive tissues, which would indicate, besides an endocrine function, also autocrine or paracrine effects (Reverchon *et al.* 2014). In this study, the putative uterine origin of ADIPOQ was addressed through the evaluation of gene transcription in the cellular pellet of uterine fluid and the endometrial protein immunolocalisation. Transcription of *ADIPOQ*, *ADIPOR1* and *ADIPOR2* was detected in the cellular pellet of uterine flushings, and persistent endometritis increased local transcription of *ADIPOQ* and its receptors. This may indicate that the presence of ADIPOQ in the uterine lumen is at least partially explained by local transcription. These findings were confirmed by the immunolocalisation of ADIPOQ, ADIPOR1 and ADIPOR2 in the cow endometrium and by the increased immunostaining of these proteins in persistent endometritis cows. Overall, our data indicate that the ADIPOQ signalling system is present in all types of endometrial cells and is related to the inflammatory status of the uterus.

A relationship between ADIPOQ concentrations and the inflammatory status was also observed in the mammary gland of cows following a lipopolysaccharide (LPS) challenge (Singh 2014) and in human patients with inflammatory and immune-mediated diseases (Fantuzzi 2013). ADIPOQ may express anti-inflammatory properties by inducing the switch of macrophage phenotype to an anti-inflammatory state, decreasing the expression of Toll-like receptor 4 (TLR4) and by suppressing the endothelial inflammatory response (Fang & Judd 2018). The cow innate immune response to LPS is dependent on TLR4 signalling in the epithelial and stromal cells of the endometrium (Herath *et al.* 2006, Sheldon & Roberts 2010, Cronin *et al.* 2012, Piras *et al.* 2017). In humans, increased TLR4 expression levels were associated with disease progression in patients with chronic endometritis (Ju *et al.* 2014), and in the bitch, pyometra caused by *Escherichia coli*, lead to a significantly increased *TLR4* transcription and protein expression in all endometrial compartments, including inflammatory cells (Silva *et al.* 2010). Therefore, endometrial *cis* and/or *trans* ADIPOQ signalling may be up-regulated following LPS injury, which may convey early predictive information regarding the establishment of endometritis. Expression of ADIPOQ and its receptors in endometrial endothelial cells is also increased in persistent endometritis cows. Therefore, the anti-inflammatory action of ADIPOQ may be also related with the suppression of the endothelial inflammatory response (Fang & Judd 2018).

This study also evidenced that cows with persistent endometritis exhibited higher plasma and uterine fluid concentrations of RARRES2 at 45 DPP than healthy and transient endometritis cows. Consistent with this, cows with persistent endometritis showed up-regulated transcription of *RARRES2*, *CMKLR1* and *GPR1* in the cellular pellet of uterine fluid and an increased expression of RARRES2 and CMKLR1 in the endometrium. These data indicate a relationship between RARRES2 signalling and the inflammatory status of the postpartum cow uterus and a local production of this mediator. However, unlike ADIPOQ, RARRES2 ROC curve and OR analysis revealed that only uterine fluid RARRES2 concentrations were suitable to discriminate persistent endometritis cows at 45 DPP. In fact, cows with uterine fluid concentrations at 45 DPP above the cutoff were a minimum of 33.3 times more at risk of evidencing endometritis at 45 DPP than healthy cows, and concentrations above the cutoff were able to discriminate all healthy from persistent endometritis cows at 45 DPP. Although adipose tissue and liver are considered the major sites of RARRES2 expression, its production has been detected in other tissues including the reproductive tract (Bongrani *et al.* 2019). This protein is expressed in several epithelial cell types and participates in the host defence mechanisms, as a broad-spectrum antimicrobial protein and as a leukocyte attractant, evidencing pro-inflammatory properties (Zabel *et al.* 2014). In fact, local RARRES2 concentrations were positively associated with metabolic and inflammatory diseases (Dranse *et al.* 2015). A systemic repercussion of a local inflammatory status, as evidenced in this study, is consistent with data from Weigert *et al.* (2010) showing increased levels of serum RARRES2 in human patients affected by inflammatory conditions.

Chemerin (RARRES2) is secreted as a precursor termed prochemerin, which is converted into the active form under conditions such as coagulation, fibrinolysis, inflammatory and complement cascades activation (Zabel *et al.* 2014). These conditions are present in the uterine lumen of cows affected with endometritis, due to the presence of PMNs. These inflammatory cells are the first to be recruited and once activated degranulate and unleash a set of proteases capable of activating prochemerin into RARRES2 (Mariani & Roncucci 2015). The observed significant positive correlation between RARRES2 concentrations in the uterine fluid, and the endometrial cytology PMN percentage at 45 DPP, is in accordance with the previously mentioned mechanism. Also, the lack of correlation between plasma and uterine concentrations of RARRES2 at 45 DPP is in accordance with a local activation and inactivation of RARRES2 by proteolytic processes (Bondue *et al.* 2011). The immunolocalisation of RARRES2 in the endometrial luminal and glandular epithelium as well as in the stroma and inflammatory cells lead to the suggestion that RARRES2 also act in the cow endometrium as a player of the pro-inflammatory cascade.

Its receptor CMKLR1 is expressed in the luminal epithelium, either at the apical membrane (groups HHP and EH) or throughout the cytoplasm (group EE). As observed in other scenarios (Zhou *et al.* 2014, De Henau *et al.* 2016), this may reflect the receptor’s internalisation after binding with RARRES2, which appeared here more pronounced in cows with persistent endometritis than in other groups. Plasma and uterine fluid NAMPT concentrations were similar in healthy and persistent endometritis cows. This finding was unexpected since NAMPT was suggested as an indicator of inflammatory disease in cows (Fadden & Bobe 2016) and to be involved in the modulation of the uterine LPS-induced inflammatory response in rats (Yang *et al.* 2015).

A significant positive correlation between ADIPOQ and RARRES2 concentrations in the uterine fluid was observed. This may represent the two faces of a balanced inflammatory response, combining anti-inflammatory (ADIPOQ) and pro-inflammatory (RARRES2) characteristics. This could act through a mechanism by which ADIPOQ could induce CMKLR1 expression and, consequently, activation of the RARRES2/CMKLR1 system, as proposed in humans following reduction of weight (Shin *et al.* 2017).

## Conclusions

In conclusion, postpartum dairy cows affected with persistent endometritis presented increased plasma and uterine fluid concentrations of ADIPOQ and RARRES2, up-regulation of transcription of *ADIPOQ*, *ADIPOR1*, *ADIPOR2*, *RARRES2*, *CMKLR1* and *GPR1* in the cellular pellet of uterine fluid, and increased expression of ADIPOQ, ADIPOR1, ADIPOR2, RARRES2 and CMKLR1 in the endometrium. Plasma and uterine fluid ADIPOQ and RARRES2 concentrations were positively correlated with the endometrial cytology PMN percentage, and the ROC curve and OR analysis showed that both plasma and uterine fluid ADIPOQ concentrations and uterine fluid RARRES2 concentrations were able to identify a persistent endometrial inflammation at 45 DPP. These data indicate a relationship between adipokine signalling and the inflammatory status of the postpartum uterus of dairy cows, where ADIPOQ and RARRES2 systems are potentially involved in a balanced anti-inflammatory and pro-inflammatory response, respectively. Therefore, ADIPOQ and RARRES2 represent suitable biomarkers able to provide an early diagnosis of subclinical endometritis and predict the risk of persistence of uterine inflammation. This would be of particular interest for discriminating cows elective for timely therapeutic approaches, as affected cows could be identified early in the postpartum period and allocated to appropriate therapy. Further studies are necessary to determine the role of these adipokines in the establishment of subclinical endometritis.

## Supplementary Material

Supplementary Table S1 - Primary antibodies.

Supplementary Table S2 – Purity and quality of RNA samples (assessed by NanoDrop Spectrophotometer and Agilent Bioanalyzer 2100) of the cellular pellets from uterine flushing used in the qPCR.

Supplementary Table S3 - Oligonucleotide primer sequences.

Supplementary Table S4 - Data distribution for analysed variables.

Suplementary Table S5 - Agreement between the vaginal discharge Metricheck score and the endometrial cytology PMN percentage at 21 (A) and 45 (B) days postpartum in dairy cows (n = 49).

Supplementary Table S6 - Plasma and uterine fluid concentrations of ADIPOQ, RARRES2 and NAMPT in healthy cows (group HH), cows with cytological endometritis at 21 DPP but recovered by 45 DPP (group EH), and cows with persistent cytological endometritis until 45 DPP.

Supplementary Table S7 - Ability of adipokines to discriminate cows with persistent cytological endometritis at 45 DPP.

## Declaration of interest

Joëlle Dupont is on the editorial board of *Reproduction*. Joëlle Dupont was not involved in the review or editorial process for this paper, on which she is listed as an author. The other authors have nothing to disclose.

## Funding

Gonçalo Pereira is a PhD student supported by a grant from Fundação para a Ciência e Tecnologia (FCT) (SFRH/BD/130,923/2017). Elisabete Silva is funded by FCT (DL 57/2016/CP1438/CT0001). This work was supported by FCT (Project UIDP/CVT/00,276/2020) and FORMAS (Grant No 2015-00,888).

## Author contribution statement

G P, P H and L L-d-C designed the study. G P, R B, J C S and L L-d-C conducted clinical evaluations and collected samples. G P, E S, C R and J D performed lab assays (analytical, ELISA, qPCR and IHC assays). Y G participated in the IHC study. G P, E S, J D, P H and L L-d-C participated in data analysis and manuscript preparation. All authors read and approved the final manuscript. P H and L L-d-C are the shared senior authors.
