# Supplementary material for: Adipokines as biomarkers of postpartum subclinical endometritis in dairy cows
Source: Reproduction. 2020 Jun 18;160(3):417–30. doi: 10.1530/REP-20-0183 (PMC7424352; doi:10.1530/REP-20-0183)
Supplement: Supplementary Table S1 - Primary antibodies. [file supplementary_table_1.pdf]

Supplementary Table S1 - Primary antibodies.

**Antibody name**	**Source**	**Dilution**	**Source** (catalogue no.)
ADIPOQ	Rabbit polyclonal	1:100	Homemade as described in Giesy et al. (2012)
ADIPOR1	Rabbit polyclonal	1:50	Antibodies-online GmbH., Aachen, Germany (ABIN2789457)
ADIPOR2	Rabbit polyclonal	1:50	Antibodies-online GmbH., Aachen, Germany (ABIN2788598)
RARRES2	Rabbit polyclonal	1:100	Santa Cruz Biotechnology as described Reverchon et al. (2014b)
CMKLR1	Mouse monoclonal	1:100	Santa Cruz Biotechnology Inc., Santa Cruz, CA, USA (sc-374570)
Rabbit IgG	Rabbit polyclonal - Isotype Control	1:200	Abcam, Cambridge, UK (ab27478)
Mouse IgG	Mouse polyclonal	1:200	Sigma-Aldrich (Ref PP54)
