# Supplementary material for: Adipokines as biomarkers of postpartum subclinical endometritis in dairy cows
Source: Reproduction. 2020 Jun 18;160(3):417–30. doi: 10.1530/REP-20-0183 (PMC7424352; doi:10.1530/REP-20-0183)
Supplement: Supplementary Table S2 – Purity and quality of RNA samples (assessed by NanoDrop Spectrophotometer and Agilent Bioanalyzer 2100) of the cellular pellets from uterine flushing used in the qPCR. [file supplementary_table_2.pdf]

Supplementary Table S2 – Purity and quality of RNA samples (assessed by NanoDrop Spectrophotometer and Agilent Bioanalyzer 2100) of the cellular pellets from uterine flushing used in the qPCR.

**Group**	***Cow ID***	***260/280 ratio***	***RIN value***
HHP	4463	1.85	8.2
HHP	5645	1.90	7.5
HHP	6450	1.75	7.6
HHP	6480	1.85	8.1
HHP	6460	1.80	7.6
HHP	3030	1.90	7.8
EH	5666	1.75	7.6
EH	5614	1.80	7.8
EH	5724	1.85	8.0
EH	3106	1.90	8.2
EH	5663	1.75	7.9
EH	5626	1.70	7.5
EH	6487	1.80	7.2
EH	6494	1.85	7.6
EH	6492	1.90	8.0
EH	6439	1.70	7.8
EH	271	1.85	8.3
EH	6540	1.90	8.5
EH	6545	1.85	8.0
EH	5707	1.80	7.5
EH	5771	1.90	8.4
EH	2141	1.85	7.9
EH	5780	1.80	8.5
EH	5805	1.75	8.0
EH	6602	1.90	7.8
EE	4454	1.85	7.7
EE	4423	1.75	7.6
EE	5638	1.70	8.0
EE	6523	1.70	7.6
EE	5734	1.85	7.5
EE	5703	1.90	7.6
EE	6575	1.90	7.8
EE	5777	1.85	7.5
EE	6608	1.80	7.4
EE	2297	1.75	7.8
EE	2333	1.80	8.1

HHP - subset of healthy cows pregnant at first AI.

EH - cows with cytological endometritis at day 21 postpartum but that recovered by day 45 postpartum.

EE - cows with persistent cytological endometritis until day 45 postpartum.

RIN - RNA integrity number.
