# Supplementary material for: Adipokines as biomarkers of postpartum subclinical endometritis in dairy cows
Source: Reproduction. 2020 Jun 18;160(3):417–30. doi: 10.1530/REP-20-0183 (PMC7424352; doi:10.1530/REP-20-0183)
Supplement: Supplementary Table S3 - Oligonucleotide primer sequences. [file supplementary_table_3.pdf]

Supplementary Table S3 - Oligonucleotide primer sequences.

Abbreviation	Name of the gene	Primer sequence 5′–3′	GenBank accession no.	Size (bp)
ACTB	Actin Beta	For: ACGGAACCACAGTTTATCATC<br>Rev: GTCCCAGTCTTCAACTATACC	D12816	188
GAPDH	Glyceraldehyde 3-phosphate dehydrogenase	For: TTCAACGGCACAGTCAAGG<br>Rev: ACATACTCAGCACCAGCATCAC	NM_001034034	119
PPIA	Cyclophilin A	For: GCATACAGGTCCTGGCATCT<br>Rev: TGTCCACAGTCAGCAATGGT	NM_178320	217
RARRES2	Chemerin	For: GAGGAGTTCCACAAGCATC<br>Rev: ACCTGAGTCTGTATGGGACA	XM_027538675.1	265
CMKLR1	Chemerin Chemokine-Like Receptor 1	For: CGGCCATGTGCAAGATCAGC<br>Rev: CAGGCTGAAGTTGTTAAAGC	XM_027566482.1	359
GPR1	G Protein-Coupled Receptor 1	For: CTGTCATTTGGTTCACAGGA<br>Rev: AACAACCTGAGGTCCACATC	MK860765.1	629
CCRL2	C-C chemokine receptor-like 2	For: AATTACACGCCAGCACCAGA<br>Rev: AGTCCTTTATATTTTACCAG	NM_001075732	199
ADIPOQ	Adiponectin	For: CACCTTCACAGGCTTCCTTC<br>Rev: AGACTGTCCTGGGAACATGG	NM_174742	219
ADIPOR1	Adiponectin receptor 1	For: GGCTCTACTACTCCTTCTAC<br>Rev: ACACCCCTGCTCTTGTCTG	NM_001034055	144
ADIPOR2	Adiponectin receptor 2	For: GGCAACATCTGGACACATC<br>Rev: CTGGAGACCCCTTCTGAG	NM_001040499	200
