# Supplementary material for: Adipokines as biomarkers of postpartum subclinical endometritis in dairy cows
Source: Reproduction. 2020 Jun 18;160(3):417–30. doi: 10.1530/REP-20-0183 (PMC7424352; doi:10.1530/REP-20-0183)
Supplement: Supplementary Table S4 - Data distribution for analysed variables. [file supplementary_table_4.pdf]

Supplementary Table S4 - Data distribution for analysed variables.

Variable	n	Shapiro-Wilk test	Distribution
Lactation number	49	< 0.001	Non Normal
Body Weight Loss Calving – 21 DPP	49	0.008	Non Normal
Body Weight Loss Calving – 45 DPP	49	0.286	Normal
Body Weight Loss Calving – 60 DPP	49	0.035	Non Normal
Milk yield by 21 DPP	49	0.461	Normal
Milk yield by 45 DPP	49	0.721	Normal
Milk yield by 60 DPP	49	0.814	Normal
NEFA at 21 DPP	49	< 0.001	Non Normal
NEFA at 45 DPP	49	< 0.001	Non Normal
Metricheck Score at 21 DPP	49	< 0.001	Non Normal
Metricheck Score at 45 DPP	49	< 0.001	Non Normal
PMN % in Cytobrush at 21 DPP	49	< 0.001	Non Normal
PMN % in Cytobrush at 45 DPP	49	< 0.001	Non Normal
Calving – first AI interval	49	< 0.001	Non Normal
Calving – conception interval	40	0.001	Non Normal
AI number/conception	40	0.001	Non Normal
Plasma Adiponectin at 21 DPP	49	< 0.001	Non Normal
Plasma Adiponectin at 45 DPP	49	< 0.001	Non Normal
Uterine fluid Adiponectin at 45 DPP	49	< 0.001	Non Normal
Plasma Chemerin at 21 DPP	49	0.110	Normal
Plasma Chemerin at 45 DPP	49	0.048	Non Normal
Uterine fluid Chemerin at 45 DPP	49	< 0.001	Non Normal
Plasma Visfatin at 21 DPP	49	0.036	Non Normal
Plasma Visfatin at 45 DPP	49	0.079	Normal
Uterine fluid Visfatin at 45 DPP	49	< 0.001	Non Normal
Standardized ADIPOQ mRNA	36	< 0.001	Non Normal
Standardized ADIPOR1 mRNA	36	0.027	Non Normal
Standardized ADIPOR2 mRNA	36	< 0.001	Non Normal
Standardized RARRES2 mRNA	36	< 0.001	Non Normal
Standardized CMKLR1 mRNA	36	< 0.001	Non Normal
Standardized GPR1 mRNA	36	0.008	Non Normal
Standardized CCRL2 mRNA	36	0.418	Normal

DPP – days postpartum
