# Supplementary material for: Adipokines as biomarkers of postpartum subclinical endometritis in dairy cows
Source: Reproduction. 2020 Jun 18;160(3):417–30. doi: 10.1530/REP-20-0183 (PMC7424352; doi:10.1530/REP-20-0183)
Supplement: Suplementary Table S5 - Agreement between the vaginal discharge Metricheck score and the endometrial cytology PMN percentage at 21 (A) and 45 (B) days postpartum in dairy cows (n = 49). [file supplementary_table_5.pdf]

Suplementary Table S5 - Agreement between the vaginal discharge Metricheck score and the endometrial cytology PMN percentage at 21 (A) and 45 (B) days postpartum in dairy cows (n = 49).

A

**Cytobrush**	**Metricheck**		**Total**
	**Positive**	**Negative**	
**Positive**	25 (51.0 %)	5 (10.2 %)	30 (61.2 %)
**Negative**	7 (14.3 %)	12 (24.5 %)	19 (38.8 %)
**Total**	32 (65.3 %)	17 (34.7 %)	49 (100.0 %)

B

**Cytobrush**	**Metricheck**		**Total**
	**Positive**	**Negative**	
**Positive**	1 (2.0 %)	10 (20.4 %)	11 (22.4 %)
**Negative**	7 (14.3 %)	31 (63.3 %)	38 (77.6 %)
**Total**	8 (16.3 %)	41 (83.7 %)	49 (100.0 %)

Metricheck score considered positive when vaginal discharge score ≥ 1.
