# Supplementary material for: Adipokines as biomarkers of postpartum subclinical endometritis in dairy cows
Source: Reproduction. 2020 Jun 18;160(3):417–30. doi: 10.1530/REP-20-0183 (PMC7424352; doi:10.1530/REP-20-0183)
Supplement: Supplementary Table S6 - Plasma and uterine fluid concentrations of ADIPOQ, RARRES2 and NAMPT in healthy cows (group HH), cows with cytological endometritis at 21 DPP but recovered by 45 DPP (group EH), and cows with persistent cytological endometritis until 45 DPP. [file supplementary_table_6.pdf]

Supplementary Table S6 - Plasma and uterine fluid concentrations of ADIPOQ, RARRES2 and NAMPT in healthy cows (group HH), cows with cytological endometritis at 21 DPP but recovered by 45 DPP (group EH), and cows with persistent cytological endometritis until 45 DPP.

**Parameters**	**HH (n = 19)**	**EH (n = 19)**	**EE (n = 11)**
Plasma ADIPOQ at 21 DPP (µg/mL)*	5.46<br>(4.90 – 6.23) [a]	4.72<br>(4.70 - 5.21) [a]	6.34<br>(6.03 - 7.43) [b]
Plasma ADIPOQ at 45 DPP (µg/mL)*	4.72<br>(4.69 – 5.53) [a]	4.56<br>(4.44 - 4.64) [b]	7.24<br>(6.81 - 7.49) [c]
Uterine fluid ADIPOQ at 45 DPP (µg/mL)*	4.71<br>(4.55 – 4.72) [a]	4.54<br>(4.32 - 4.57) [a]	10.39<br>(10.19 - 10.56) [b]
Plasma RARRES2 at 21 DPP (ng/mL)**	2.47 ± 0.05	2.60 ± 0.06	2.48 ± 0.07
Plasma RARRES2 at 45 DPP (ng/mL)*	2.44<br>(2.36 - 2.56) [a]	2.56<br>(2.31 - 2.65) [a,b]	2.63<br>(2.59 - 2.64) [b]
Uterine fluid RARRES2 at 45 DPP (ng/mL)*	1.49<br>(1.43 - 1.50) [a]	1.42<br>(1.34 - 1.44) [a]	3.07<br>(2.91 - 3.38) [b]
Plasma NAMPT at 21 DPP (ng/mL)*	155.93<br>(129.50 – 166.17) [a,b]	148.07<br>(140.21 – 155.69) [a]	174.5<br>(153.31 – 200.93) [b]
Plasma NAMPT at 45 DPP (ng/mL)**	171.35 ± 4.89 [a]	150.84 ± 3.45 [b]	184.98 ± 5.94 [a]
Uterine fluid NAMPT at 45 DPP (ng/mL)*	138.07<br>(135.21 – 148.07)	153.31<br>(137.36 - 155.93)	137.36<br>(121.17 – 155.93)

DPP = days postpartum

*Values reported as median and (interquartile range) for non-normally distributed data.

**Values reported as mean ± SEM for normally distributed data.

Different letters indicate significant differences between groups (level of significance *P* < 0.05) determined with ANOVA and the non-parametric Kruskal-Wallis-Test with Dunns post-test for normally and non-normally distributed variables, respectively.
