# Supplementary material for: Adipokines as biomarkers of postpartum subclinical endometritis in dairy cows
Source: Reproduction. 2020 Jun 18;160(3):417–30. doi: 10.1530/REP-20-0183 (PMC7424352; doi:10.1530/REP-20-0183)
Supplement: Supplementary Table S7 - Ability of adipokines to discriminate cows with persistent cytological endometritis at 45 DPP. [file supplementary_table_7.pdf]

Supplementary Table S7 - Ability of adipokines to discriminate cows with persistent cytological endometritis at 45 DPP.

**Adipokines**	**Number of cows under the cutoff value**			**Number of cows above the cutoff value**			**OR**	**OR [CI 95%]**	***P* value**
	**Healthy**	**Endometritis**	**Total**	**Healthy**	**Endometritis**	**Total**			
Plasma ADIPOQ at 21 DPP (cutoff: 5.9 µg/mL)	31	2	33	7	9	16	19.9	3.5 to 113.3	0.0007
Plasma ADIPOQ at 45 DPP (cutoff: 6.1 µg/mL)	38	1	39	0	10	10	539	20.4 to 14218.7	0.0002
Uterine fluid ADIPOQ at 45 DPP (cutoff: 7.8 µg/mL)	38	0	38	0	11	11	1771	33.3 to 94301.3	0.0002
Plasma RARRES2 at 21 DPP (cutoff: 2.2 ng/mL)	4	0	4	34	11	45	3	0.15 to 60.1	0.4725
Plasma RARRES2 at 45 DPP (cutoff: 2.5 ng/mL)	19	0	19	19	11	30	23	1.3 to 418.1	0.0341
Uterine fluid RARRES2 at 45 DPP (cutoff: 2.2 ng/mL)	38	0	38	0	11	11	1771	33.3 to 94301.3	0.0002

DPP = days postpartum; OR = odds ratio; CI = confidence interval.
